# Understanding the Risk Factors and Pathogenesis of Disseminated Nocardiosis in Immunocompromised Patients

**DOI:** 10.7759/cureus.59838

**Published:** 2024-05-07

**Authors:** Kacper Bury, Vincent Citriniti, Sheeva Bahrampour, Sonal Bajaj, John F Ferguson

**Affiliations:** 1 Internal Medicine, Touro College of Osteopathic Medicine, Middletown, USA; 2 Infectious Disease, Garnet Health Medical Center, Middletown, USA; 3 Pulmonology, Garnet Health Medical Center, Middletown, USA

**Keywords:** lung nocardiosis, cerebral nocardiosis, infection, brain abscess, disseminated nocardiosis, immunocompromised, nocardia

## Abstract

*Nocardia *is a genus of aerobic, Gram-positive, partially acid-fast, filamentous bacilli notoriously known for causing multisystemic infections in immunocompromised individuals. Notably, this genus of bacteria commonly infects the pleural and central nervous system, leading to pneumonia and brain abscesses, respectively. Our patient is a 71-year-old female who initially presented to the emergency department complaining of shortness of breath and altered mental status. Imaging revealed multiple enhancing brain lesions, a pleural effusion, and a paraspinal abscess, which upon aspiration and culture demonstrated *Nocardia farcinica/kroppenstedtii*. The patient underwent antibiotic treatment, including intravenous (IV) imipenem and trimethoprim/sulfamethoxazole (TMP-SMX), before being transitioned to oral TMP-SMX and amoxicillin/clavulanate. This case demonstrates the importance of diagnosing nocardiosis acutely and treating it appropriately.

## Introduction

The *Nocardia* genus consists of aerobic bacteria that are Gram-positive, partially acid-fast, and catalase-positive. These filamentous bacilli were first found in cattle infected with Farcy disease, which causes granulomatous abscess formation and pulmonary symptoms [1]. It is hypothesized that the *Nocardia* genus is widely infective to several species of animals including fish, horses, cats, dogs, and humans due to its guanine-cytosine-rich genome [1].

According to the Centers for Disease Control (CDC), nocardiosis has an incidence of 500-1000 cases per year in the US, which experts predict will gradually increase over the years due to an increase in immunocompromised patients [2]. Nocardia is classically an opportunistic infection that may infect individuals who have respiratory or immunological illnesses. Typically, these patients will initially present with pulmonary symptoms and cavitation. As *Nocardia* progresses, the patient may present with central nervous system (CNS) symptoms secondary to brain abscesses because of bacteria’s high affinity for neural tissue. The patient described in the following case report is a 71-year-old female with an extensive past medical history, most notably, chronic respiratory failure post-COVID-19 infection, who subsequently developed disseminated nocardiosis presenting as multiple CNS abscesses, soft tissue abscesses, and pulmonary disease.

## Case presentation

**Figure 1:**
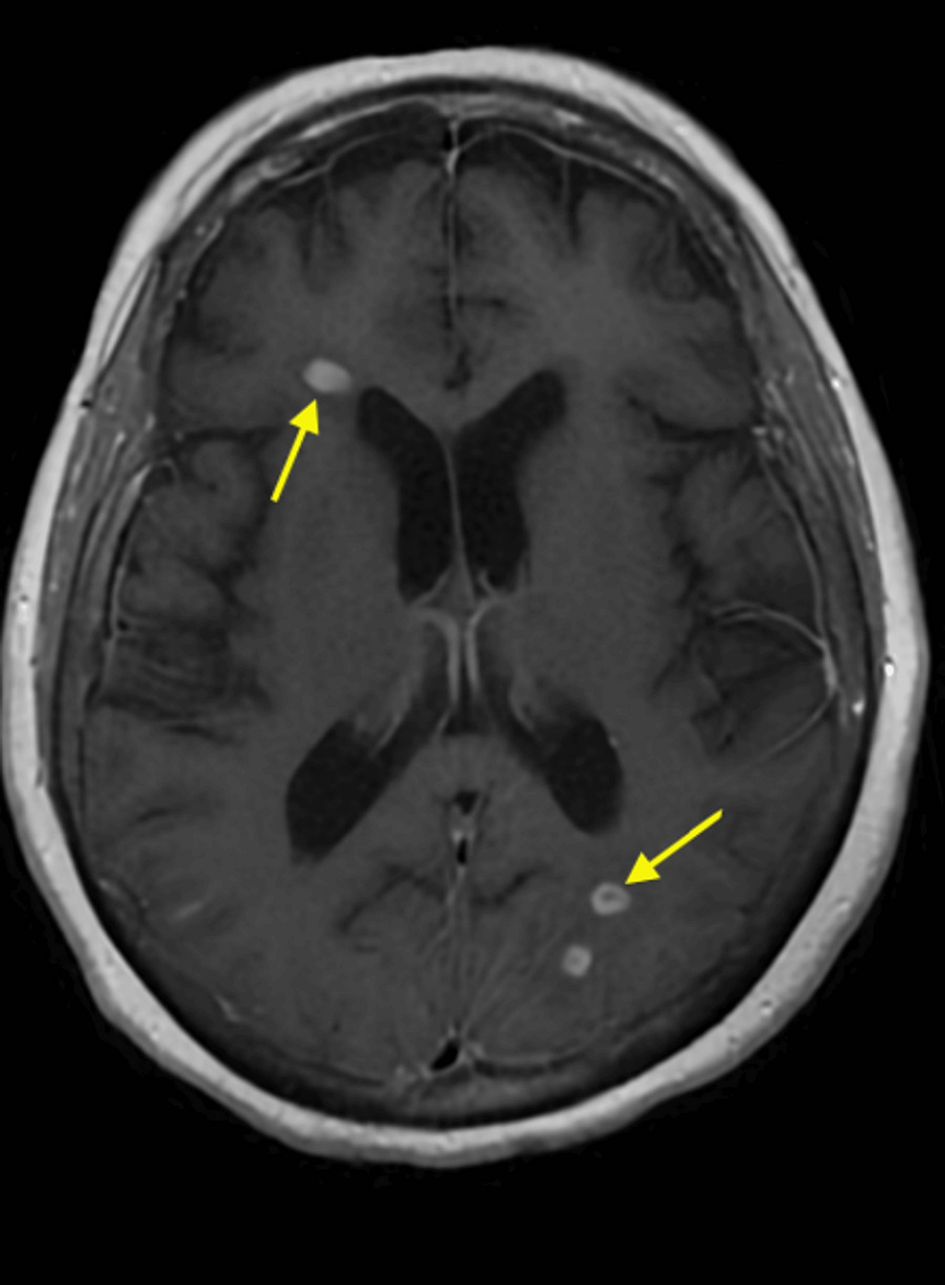
Magnetic resonance imaging (MRI) showing multiple brain abscesses

**Figure 2:**
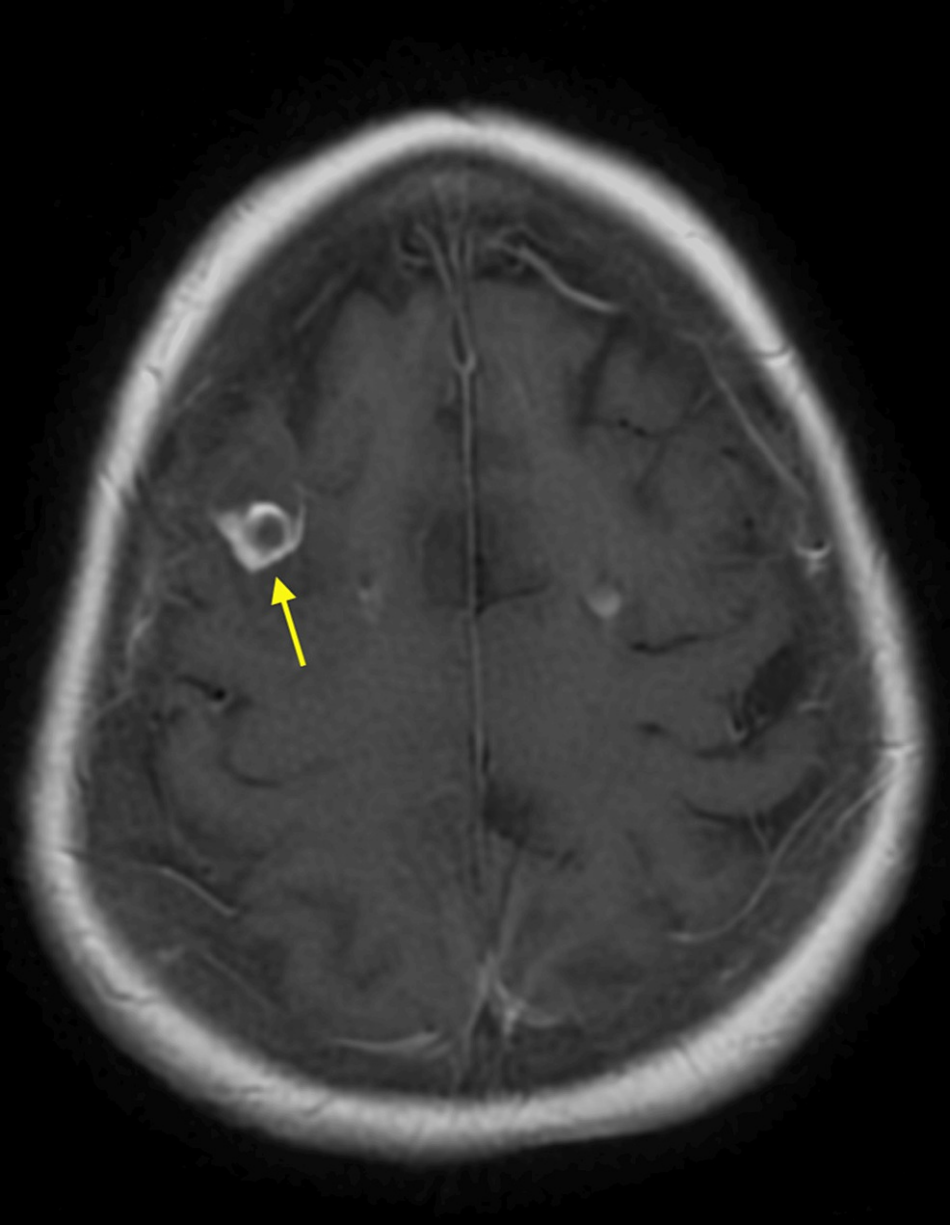
Magnetic resonance imaging (MRI) showing a ring enhancing lesion

A 71-year-old female with a past medical history of chronic obstructive pulmonary disease (COPD), asthma, diabetes mellitus type 2, hypertension, depression, anemia, right-sided hemi-diaphragmatic paralysis, chronic respiratory failure with hypoxia post-COVID-19 infection, and oxygen dependence due to COPD presented to the emergency department (ED) with a complaint of worsening shortness of breath, productive cough, altered mental status, and decreased appetite. The patient was transferred from a primary care facility to a community hospital ED due to multiple peripherally enhancing brain lesions found on a magnetic resonance imaging (MRI) scan of the brain (Figures 1, 2).

**Figure 3:**
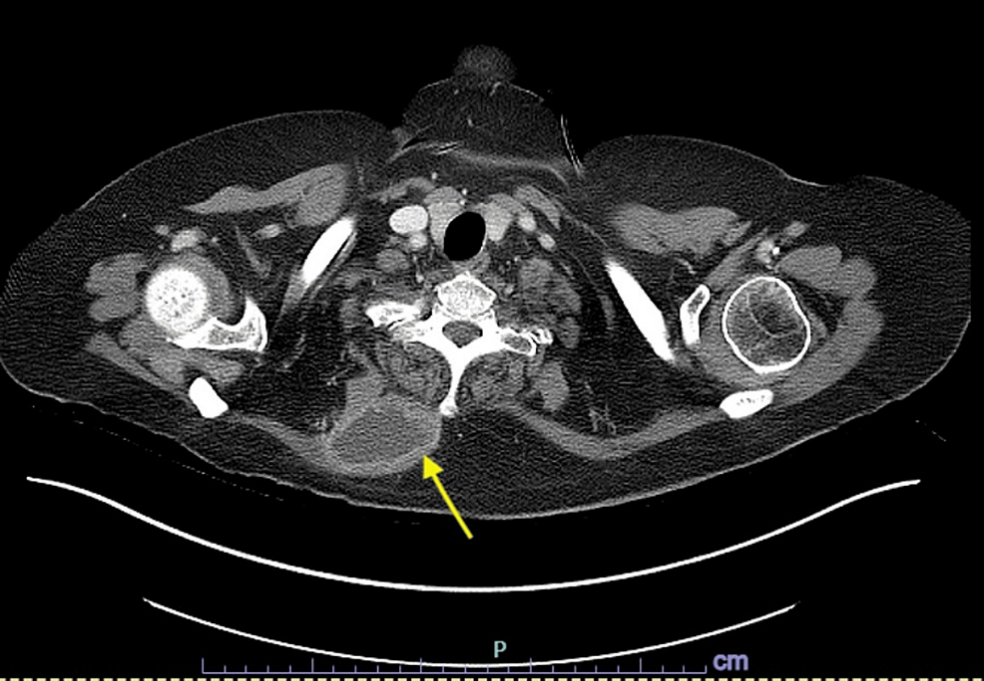
Computed tomography (CT) of the chest showed a soft tissue mass The patient's CT of the chest with contrast showed a right paraspinal abscess (yellow arrow) measuring 5.5 x 3.8 x 5.5 cm

**Figure 4:**
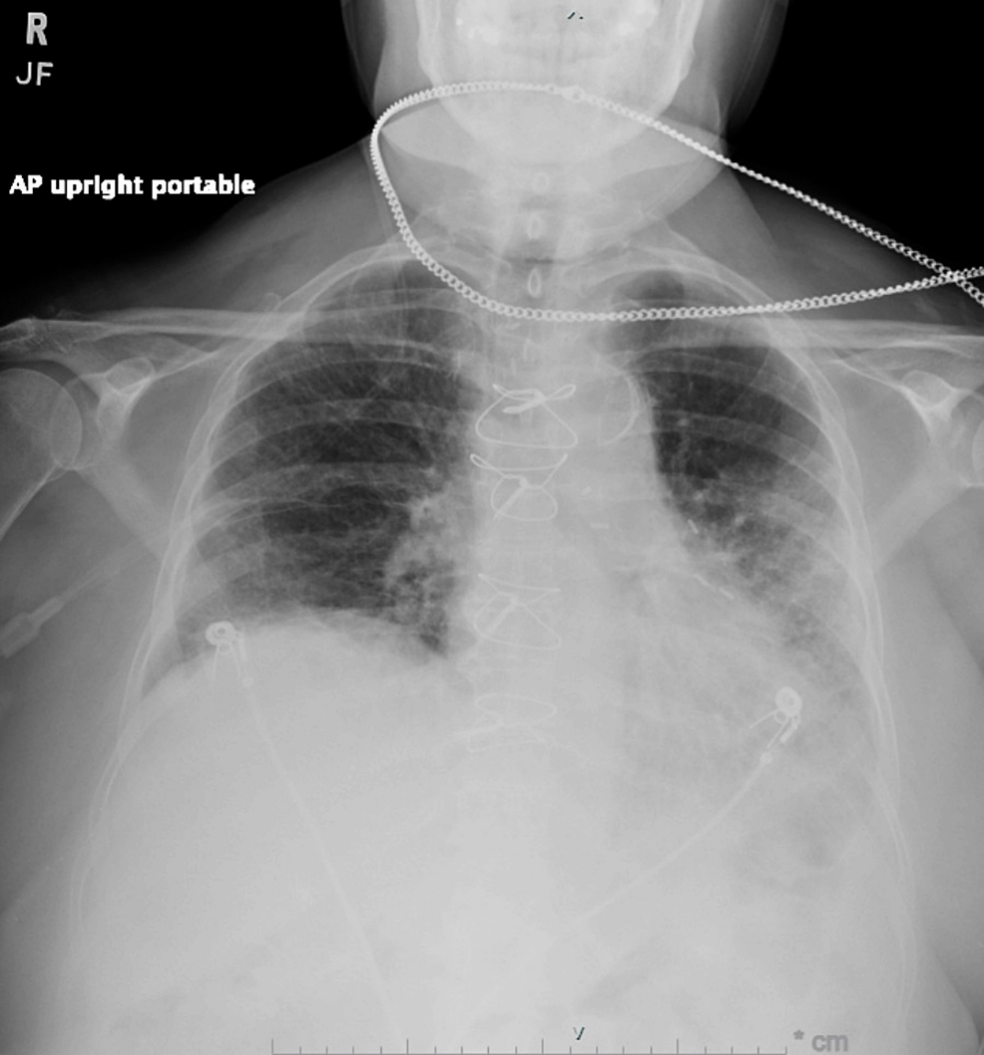
Chest x-ray The patient's chest x-ray revealed bilateral infiltrates with a small right pleural effusion

Upon arrival at the receiving facility, computerized tomography (CT) of the chest with contrast showed an enhancing right upper paraspinal mass, measuring 5.5 x 3.8 x 5.5 centimeters (cm) (Figure 3). A CT of the abdomen/pelvis revealed a left abdominal wall mass with bilateral interstitial infiltrates without honeycombing and a right small pleural effusion. The chest X-ray (CXR) showed bilateral infiltrates with a small right pleural effusion (Figure 4). The patient's 12 lead electrocardiogram (EKG) showed normal sinus rhythm with a prolonged QT interval. 

On physical examination, the patient's vital signs were as follows: temperature of 97.9℉ (36.6℃), blood pressure of 148/65, pulse of 109, respiratory rate of 31, and oxygen saturation of 87% on room air. Upon lung auscultation, bilateral lower lung rhonchi were found. The Glasgow Coma Scale (GCS) was 9 and the patient was found to have +1 bilateral lower extremity edema. The patient's lab work showed a white blood cell (WBC) count of 23.0 x10^3^/L, hemoglobin of 9.1 g/dL, and a platelet count of 680 x10^3^/L (Table 1).

**Table 1:** Laboratory findings in the emergency department CK: creatine kinase; eGFR: estimated glomerular filtration rate; ALT: alanine aminotransferase; AST: aspartate aminotransferase; BUN: blood urea nitrogen; INR: international normalised ratio ; PTT: partial thromboplastin time; MCV: mean corpuscular volume ; MCHC: mean corpuscular hemoglobin concentration ; RDW: red cell distribution width

Test	Result	Reference Range
Complete Blood Count (CBC)		
WBC	23.0 x10^3^/L	3.9 - 10.7 x10^3^/L
Hemoglobin	9.1 g/dL	12.0-14.7 g/dL
Hematocrit	30.20%	37.1-45.4%
MCV	86.8 fL	81.9-99.9 fL
MCHC	30.1 g/dL	30.9-33.6 g/dL
RDW	16.60%	11.5-15.0%
Platelets	680 x10^3^/L	126-373 x10^3^/L
Absolute Neutrophil Count	20286 cells/mm^3^	1500-8000 cells/mm^3^
Absolute Lymphocytes	1.4 x10^3^/L	0.7-3.6 x10^3^/L
Absolute Monocytes	0.8 x10^3^/L	0.2 - 1.1 x10^3^/L
Eosinophils	0.02 x10^3^/L	0.00 - 0.5 x10^3^/L
Basophils	0.1 x10^3^/L	0.0 - 0.1 x10^3^/L
Coagulation Studies		
Protime	15.3 secs	10.1-12.9 secs
INR	1.33	0.88 - 1.12
PTT	32.5 secs	25.1 - 36.5
Chemistry Studies		
Sodium	139 mEq/L	136-144 mEq/L
Potassium	3.1 mEq/L	3.5-5.1 mEq/L
Chloride	102 mEq/L	101-111 mEq/L
CO_2_	26 mEq/L	22-32 mEq/L
BUN	11 mg/dL	8-20 mg/dL
Creatinine	1.05 mg/dL	0.55-1.02 mg/dL
Total Bilirubin	0.3 mg/dL	0.3-1.2 mg/dL
Albumin	2.2 g/dL	3.5-4.8 g/dL
Alkaline Phosphatase	101 U/L	45-117 U/L
AST	35 U/L	15-41 U/L
ALT	20 U/L	14-54 U/L
Anion Gap	11 mEq/L	8-17 mEq/L
eGFR	56.8 mL/min/1.73m^2^	> 60 mL/min/1.73m^2^
Lactic Acid	1.0 mmol/L	0.5-2.2 mmol/L
Procalcitonin	0.370 ng/mL	<0.500 ng/mL
Cardiac Markers		
Total CK	19 U/L	38-234 U/L
Troponin I	<0.03 ng/mL	<0.03 ng/mL

Due to the patient's presentation, she was admitted for further evaluation and to exclude neoplasm vs an infectious process. PCRs for SARS-CoV2, and tests for influenza A and B, and RSV were negative. No growth was isolated in the urine, blood, or sputum cultures. Per recommendations given by Infectious Diseases (ID), Interventional Radiology (IR) was consulted for a biopsy of the right upper back mass. The right upper paraspinal abscess was aspirated. Gram stain revealed branching gram-positive bacilli which was highly suggestive of *Nocardia*. IV Imipenem 500 milligrams (mg) every 6 hours and IV TMP/SMX 340mg every 8 hours (15mg/kg of trimethoprim per day in 3 divided doses) were initiated based on gram stain results. Broad-range bacterial PCR and sequencing requested through the Mayo Clinic identified *Nocardia* species. This isolate was identified as *Nocardia farcinica/kroppenstedtii.*

On day 23 of IV therapy, the patient developed lactic acidosis, thought to be secondary to the accumulation of propylene glycol in the TMP/SMX formulation. The patient was then adjusted to oral TMP/SMX which resolved the lactic acidosis. After 53 days of IV imipenem, the patient was transitioned to oral amoxicillin/clavulanate while oral TMP/SMX was discontinued. Oral TMP/SMX had to be discontinued as the patient developed nephrolithiasis secondary to sulfamethoxazole. Oral amoxicillin/clavulanate monotherapy was continued with excellent response. At 9 months, all previously seen cerebral, pleural, and soft tissue abscesses were resolved.

## Discussion

The patient's past medical history is of great importance to her current immunocompromised state. Nearly two years before her *Nocardia* infection, the patient developed respiratory failure with hypoxia secondary to COVID-19. She presented with a cough, dizziness, and worsening shortness of breath. She was treated initially with dexamethasone 10mg IV twice a day, and monoclonal antibodies. Following treatment for her respiratory failure, a CT scan of the chest showed bilateral ground glass opacities. Due to her history and presentation at the time, the patient was admitted to the hospital where she continued to be treated. The patient was placed on her at-home oxygen therapy at 2-4 L/min. The patient then developed glucocorticoid-induced diabetes. Her new diabetes diagnosis further contributed to her immunocompromised state.

Twenty-two months before contracting Nocardia, the patient underwent cardiac catheterization in the setting of ongoing fatigue, weakness with minimal exertion, shortness of breath, and a CT calcium score of 524. The cardiac catheterization found 50% occlusion of the left marginal, distal left anterior descending, and left circumflex arteries, along with an ejection fraction of 40-45%. As a result, the patient was diagnosed with cardiomyopathy. A month later, the patient underwent coronary artery bypass surgery (CABG). Unfortunately, the surgery was complicated by injury to an innominate vein and artery requiring chest exploration and repair of the innominate vessels. During this hospitalization, she was found to have an injury to the right phrenic nerve with associated right hemidiaphragm paralysis. Additionally, the patient began treatment with antibiotics for unspecified anterior cutaneous ulcers in her right lower extremity. As a result of these complications during her hospitalizations, the patient was sent to pulmonary rehabilitation for three months and required 5L of at-home oxygen. It is postulated that the patient’s extensive medical history, along with her prolonged hospitalization, led the patient to contract pneumonia secondary to *Nocardia farcinica* infection, and subsequently disseminated nocardiosis. 

The mechanism of action by which *Nocardia* infects the host is also facilitated by an immunocompromised state. As stated earlier, *Nocardia* is a branching, gram-positive rod that belongs to the aerobic actinomycetes group [1]. *Nocardia* can infect the host either by aerosolized droplets or through direct skin inoculation [3]. Unfortunately, this patient's disease course and multiple hospital complications have made it difficult to pinpoint where and when exactly her infection may have originated. The patient had multiple pulmonary morbidities, including COPD and chronic respiratory failure due to COVID-19. This may have resulted in droplet transmission of *Nocardia* during her extended hospital course. Another source of infection could have been the patient's lower extremity cutaneous ulcers. There have been previous incidences of cutaneous *Nocardia* evolving into a systemic infection, but most patients suffer first from pulmonary nocardiosis before it spreads systemically and forms cutaneous ulcers [4]. Primary cutaneous nocardiosis would be more likely if there were a history of penetrating trauma or exposure of preexisting wounds to contaminated soil. 

An immunocompromised patient is more susceptible to uncommon infections, particularly in the lungs. One of the primary organ systems contributing to passive and active immunity is the respiratory tract. The pulmonary system provides patients with the mucociliary apparatus that aids in physically clearing pathogens. There is literature supporting that an impairment of the cilia apparatus places patients at risk for opportunistic Nocardia infections [5]. For example, one case report highlighted an otherwise healthy 14-year-old female with a history of primary ciliary dyskinesia who presented with lobar pneumonia [5]. Her bronchoscopy with bronchoalveolar lavage and other appropriate imaging confirmed a diagnosis of Nocardial pleuropneumonia [5].

Additional research suggests that COVID-19 itself, which our patient contracted, may induce ciliary dysfunction [6]. Of note, Bjorn Afzelius, the individual who first described primary ciliary dyskinesia, detected coronavirus within ciliated cells [6]. He described that after infection with coronavirus, the cilia are not damaged, but rather are removed from the primary cell [6]. There are two models proposed for this loss of cilia. The first is called the amputation model, in which the entire cilium is eliminated at the base; the second is called the absorption model, in which the infected cell gradually absorbs the cilium [6]. Changes to this unique feature may include ciliary dysfunction, increased mucus production, and destruction of the epithelial lining [6]. Smoking is also a significant source of ciliary destruction in the airways, as described above. The reduction in size of cilia from cigarette smoke leads to decreased mucociliary clearance of inhaled pathogens and particulates [7,8]. The patient discussed in this current case report is a former smoker with an unknown number of pack years, however, she was not smoking at the time of her illness or when the COVID-19 pandemic began. Nonetheless, her smoking history has made her more susceptible to respiratory tract infections.

Literature continues to support that some viruses, such as COVID-19, are pathogenic to cilia [6]. One study demonstrated that the nasal epithelial cells underwent loss of their cilia when infected by coronavirus [6]. This may be linked to the symptomatology of anosmia in COVID-19 patients and may affect the mucociliary escalator making patients more prone to bacterial infections [6]. Other studies demonstrate that an excessive innate immune response to COVID-19, specifically the neutrophilic response, may induce a cytokine storm and lead to tissue damage [9]. Regardless of the mechanism, it is imperative to consider COVID-19 as an immuno-impairing virus that may put patients at risk for opportunistic bacterial infection. 

This patient's respiratory system continued to deteriorate due to the hemidiaphragm paralysis that arose as a complication of her cardiac surgery. The diaphragm is one of the primary muscles of inspiration, innervated by the phrenic nerve. Not only does this dome-shaped muscle help patients breathe, but it also contributes to coughing, an essential immunological mechanism. A cough is an innate reflex that protects lung tissue against foreign materials and promotes the expulsion of bacteria via the mucociliary escalator [10]. This patient’s cough reflex was severely affected by hemidiaphragmatic paralysis, which endorsed further immunosuppression and increased the likelihood of contracting a foreign bacteria or virus, such as *Nocardia*, that is transmitted through aerosolized or respiratory droplets. As a natural response to help the patient breathe, the body may have depended on accessory muscles of respiration, which are much smaller in size and cannot keep a constant inspiratory load for an extended period due to muscle fatigue [11]. Overall, respiratory muscle weakness and fatigue have been shown to correlate with higher mortality rates and increased susceptibility to lower respiratory tract infections [12,13]. This unfortunate intraoperative injury that the patient suffered resembles a restrictive lung disease. As time progressed, the compliance of the lungs and surrounding muscles decreased, which in turn led to a compromised mucociliary esclator and reduced elimination of pathogens [14].

It is also worth noting that this patient was on glucocorticoid therapy for at least one year before she presented with *Nocardia*, which contributed to the patient’s immunosuppressed state. She was on a variety of doses ranging from 10-40mg at a time, intermittently. Glucocorticoids have been noted to have broad immune system effects, particularly due to the broad distribution of glucocorticoid receptors across different cell types [15]. One study examining T cells demonstrated specifically that dexamethasone upregulated CTLA-4 mRNA and CTLA-4 protein in T cells, and blocked CD28, a costimulatory molecule found in T cells, which mediated cell cycle entry and differentiation [16]. Outside of T cells, research has also demonstrated that other cells, such as dendritic cells, are also affected, resulting in hypo-responsiveness in T cells and overproduction of Treg cells, which diminishes the immune response [16]. The effects of glucocorticoids on the immune system are widespread and robust, allowing for their exogenous use as anti-inflammatories and immunomodulators. This patient was on significant glucocorticoids before admission to the hospital. While her immunocompromised state is multifactorial, her prior history of glucocorticoid use was additive to this state, and thus put her at a higher risk of acquiring *Nocardia*. 

## Conclusions

In this case, disseminated Nocardiosis was heavily influenced by the patient’s immunocompromised state developed through a combination of her acute hypoxic respiratory failure secondary to COVID-19 in the setting of pre-existing COPD, and history of smoking with an unknown number of pack years, chronic corticosteroid treatment, newly diagnosed diabetes mellitus type 2, and hospitalization for CABG, further complicated by right phrenic nerve injury and subsequent hemidiaphragm paralysis. Given the proven association between *Nocardia* and the immunocompromised, this patient’s poor health state, as per her comorbidities and hospital complications, was critical to the progression of her disease.

Nine months after her initial diagnosis, there has been a complete resolution of all cerebral, pleural, and soft tissue-based abscesses. The patient will remain on monotherapy with oral amoxicillin/clavulanate to complete a total 12-month course, with plans for chronic antibiotic suppression, as she may require advanced monoclonal antibody treatments for her other underlying conditions.
